# Transcriptome analysis of aerotolerant and aerosensitive *Campylobacter jejuni* strains in aerobic conditions

**DOI:** 10.3389/fmicb.2025.1621314

**Published:** 2025-07-30

**Authors:** Elise Delaporte, Anand B. Karki, Mohamed K. Fakhr

**Affiliations:** ^1^Department of Biological Science, The University of Tulsa, Tulsa, OK, United States; ^2^Department of Biological Sciences, Sam Houston State University, Huntsville, TX, United States

**Keywords:** *Campylobacter*, aerotolerance, transcriptome, oxidative stress, differential gene expression

## Abstract

Aerotolerance is vital for the survival of *Campylobacter jejuni* in the food supply, but the genetic mechanisms underlying aerotolerance remain unclear. This study compares differential gene expression in one aerotolerant and one aerosensitive strain of *C. jejuni* (WP2202 and T1-21 respectively) in aerobic vs. microaerobic conditions using RNA-Seq technology. The results show that the aerotolerant strain differentially regulated a greater number of genes under aerobic vs. microaerobic conditions as compared to the aerosensitive strain, particularly during the first 6 h of exposure. Differential analysis between aerobic and microaerobic conditions showed that COG category S (genes with unknown functions) had the highest number of DEGs across all timepoints in both strains. When category S was excluded, COG category J (translation, ribosomal structure, and biogenesis) had the highest number of DEGs between aerobic vs. microaerobic conditions with downregulated genes occurring at most timepoints in the two strains. Several previously characterized oxidative stress genes were differentially regulated in both strains in response to aerobic conditions. Both strains upregulated multiple heat shock genes in response to oxygen exposure, supporting the hypothesis that these genes might play a role in the oxidative stress response. A few genes involved in iron acquisition or transport were significantly upregulated under aerobic conditions in the aerosensitive strain, potentially forming reactive oxygen radicals due to increased iron levels. A spike in gene expression after 12 h of oxygen exposure was noted for both strains in various genes across the genome. This study demonstrates differences in differential gene expression between an aerotolerant and an aerosensitive strain in response to exposure to atmospheric oxygen and sheds light into understanding *C. jejuni* aerotolerance. Numerous genes with potential roles in *C. jejuni* aerotolerance were identified which provides new avenues for future research. In particular, the benefits and drawbacks of iron to the oxidative stress response and the links between the oxidative stress response and the expression of heat shock genes require further investigation.

## 1 Introduction

Campylobacteriosis is the primary cause of bacterial diarrhea and sickens approximately 1 in 10 people annually (WHO, 2020). Campylobacteriosis generally lasts for 3–6 d, and symptoms incited by *Campylobacter* spp. include diarrhea, fever, headache, abdominal pain, nausea, and vomiting (WHO, 2020). Complications from *Campylobacter* infection can be serious and even fatal, with most fatalities occurring in the very young, elderly, and immunocompromised (WHO, 2020). Various post-infection complications are possible including arthritis, irritable bowel syndrome, and Guillain-Barré syndrome (CDC, 2024b); the latter causes muscle weakness that can progress into paralysis (CDC, 2024a). Recovery from Guillain-Barré syndrome can take months or even years with some cases leading to permanent paralysis (CDC, 2024a), and *Campylobacter* infections are one of the leading causes of the syndrome (Poropatich et al., 2010; CDC, 2024a).

One of the primary causal agents of campylobacteriosis is *Campylobacter jejuni* (WHO, 2020), which is a gram-negative, motile bacteria (Perez-Perez and Blaser, 1996). The bacteria is frequently cultured *in vitro* at 42°C, the body temperature of chickens, or 37°C, the body temperature of humans, but can survive a range of temperatures (Aroori et al., 2013; Khan et al., 2013). *C. jejuni* is microaerophilic and requires an O_2_ concentration of 3–10% for growth; however, some strains were reported to be aerotolerant (Oh et al., 2015a), which allows the bacterium to survive meat processing procedures. Strains of *Campylobacter* that are capable of surviving for more than 12 h of exposure to atmospheric oxygen levels during incubation with agitation are classified as aerotolerant, and those that survive more than 24 h are considered hyper-aerotolerant (Oh et al., 2015a). Aerotolerant *Campylobacter* spp. are highly prevalent in strains isolated from raw meat, with some studies reporting 71.4–86.6% of isolates to be aerotolerant (Oh et al., 2015a; Kim et al., 2019). Clinical isolates also exhibit aerotolerance (Oh et al., 2018), and this trait increases the likelihood of human infections. The prevalence of aerotolerant isolates in raw meat and human patients highlights the importance of studying how microaerophilic *Campylobacter* spp. develop aerotolerance and survive in atmospheric oxygen.

Despite this significance, the genetic mechanisms underlying aerotolerance in *C. jejuni* are not well understood. Genes known to be involved in aerotolerance include *katA*, *ahpC*, and *sodB*, which encode catalase, alkyl hydroperoxide reductase, and superoxide dismutase, respectively (Vercellone et al., 1990; Purdy and Park, 1994; Baillon et al., 1999). Numerous studies have demonstrated relationships between these three genes and resistance to O_2_ exposure or oxidative stress (Kikuchi and Suzuki, 1984; Grant and Park, 1995; Purdy et al., 1999; van Vliet et al., 1999; Lee et al., 2005; Handley et al., 2015; Oh et al., 2015a, 2015b, 2019; Rodrigues et al., 2016; Jeon et al., 2022). Other *Campylobacter* genes have been noted as potential contributors to aerotolerance, but most are not well-characterized and many have unknown functions (Stahl and Stintzi, 2011). Certain strategies have been implicated in *Campylobacter*’s aerotolerance as well such as biofilm formation (Joshua et al., 2006; Asakura et al., 2007; Reuter et al., 2010; Turonova et al., 2015; Feng et al., 2016; Oh et al., 2016; Kassem et al., 2017; Efimochkina et al., 2019; Zhong et al., 2020; Mouftah et al., 2021; Kanaan, 2024), the viable-but-nonculturable (VBNC) state (Moran and Upton, 1987a; Lee et al., 2005; Zhong et al., 2020), and polymicrobial interactions with other bacteria and even amoeba (Axelsson-Olsson et al., 2007; Wongjaroen et al., 2008; Hilbert et al., 2010; Bui et al., 2012; Karki et al., 2021; Anis et al., 2022; Šimunović et al., 2022). Delaporte et al. (2024) have summarized previous research on potential genetic mechanisms and other strategies which may contribute to aerotolerance (Delaporte et al., 2024).

In this study, we use RNA-Seq to compare gene expression in strains of *C. jejuni* in response to O_2_ exposure. Gene expression during microaerobic conditions (Time 0) were compared to aerobic conditions (Times 0.5, 6, 12, and 24 h) in the aerosensitive T1-21 and aerotolerant WP2202 strains of *C. jejuni*. The data were analyzed to detect differences in gene expression in response to microaerobic growth and O_2_ exposure and provide valuable information on how aerotolerant and aerosensitive *C. jejuni* strains differ in their response to oxygen exposure.

## 2 Materials and methods

### 2.1 Strain selection and initial culture conditions

In this study, we used two strains previously isolated and sequenced by our lab, namely *C. jejuni* T1-21 (GenBank accession nos. NZ_CP013116.1, NZ_CP013117.1) and *C. jejuni* WP2202 (accession nos. NZ_CP014742.1, NZ_CP014743.1) (Marasini and Fakhr, 2016a, 2016b). Based on previous aerotolerance assays conducted in our lab, the T1-21 and WP2202 strains were selected based on aerosensitivity and aerotolerance, respectively (Karki et al., 2018). The strains were cultured microaerobically (CampyGen™ 3.5 L, Thermo Scientific, Waltham, MA, United States) for 48 h at 42°C on Mueller Hinton agar made from Mueller Hinton broth (Hardy Diagnostics, Santa Maria, CA) and bacteriological agar (Oxoid, Thermo Scientific, Waltham, MA, United States) supplemented with 5% laked horse blood and Bolton Selective Supplement (Himedia), and then subcultured to Mueller Hinton agar (MHA) containing 5% laked horse blood for an additional 48 h in the same conditions. Colonies were then inoculated into 125 mL conical flasks containing 75 mL of Mueller Hinton Broth (Hardy Diagnostics, Santa Maria, CA) and incubated with agitation under microaerobic conditions for 16 h at 42°C. Cells were harvested from the broth via centrifugation and resuspended to an OD600 = 1.0. Conical flasks (125 mL) containing 20 mL fresh MHB were inoculated in triplicate with 2 mL of the bacterial suspensions and incubated an additional 1.25 h in microaerobic conditions prior to the experiments described below.

### 2.2 Sample preparation, RNA isolation, and sequencing

Per the suggested protocol for sample preparation from Zymo Research’s Directzol RNA miniprep kit, cell samples (1 mL) for time 0 were collected and centrifuged at 10,000 RPM (7,378 × g) for 2 min to obtain cell pellets in a benchtop microfuge (Beckman Coulter, Microfuge 16). Supernatants were discarded, replaced with 700 μL of TRI reagent (Zymo Research, Irvine, CA, United States), and frozen at −80°C. The samples were then incubated aerobically with agitation at 42°C and the sample collection method described above was used to collect additional samples at times 0.5, 6, 12, and 24 h. RNA was isolated using the Directzol RNA miniprep kit (Zymo Research, Irvine, CA, United States), and contaminating DNA was removed with the TURBO DNA-free kit (Invitrogen, Vilnius, Lithuania). Total RNA was quantified with a Nanodrop spectrophotometer and Qubit™ RNA HS Assay Kit (Invitrogen, Eugene, OR, United States). RNA quality was analyzed with an Agilent 2100 Bioanalyzer and the Agilent RNA 6000 Pico kit (Agilent, Santa Clara, CA, United States). A PCR assay with three selected housekeeping genes (*aspA*, *glyA*, and *16S*) was conducted to evaluate that the RNA was DNA-free. The Illumina TruSeq Stranded mRNA Library Prep kit (Illumina, San Diego, CA, United States) was used to prepare cDNA libraries using approximately 100 ng of each RNA sample. cDNA libraries were quantified with the Qubit dsDNA HS Assay kit, and DNA quality was evaluated using the Agilent Bioanalyzer 2100 and the Agilent High Sensitivity DNA kit (Agilent, Santa Clara, CA, United States). Sequencing was outsourced to a sequencing service provider (Quick Biology Inc., Monrovia, CA, United States) and was conducted with an Illumina Hiseq 4000 platform. All the original sequence reads for this experiment are accessible in the Sequence Read Archive (SRA) database within Bio project id: PRJNA1220700.

### 2.3 Data analysis

Analysis was undertaken for the purpose of differentially comparing the gene expression of each strain under the aerobic conditions (Time 0.5, 6, 12, and 24 h) to their gene expression under the microaerobic condition (Time 0). The following steps were performed to analyze sequencing data using the CLC Genomics Workbench 22 (Qiagen). Automatic read-through adaptor trimming was employed to remove the adaptors with the following settings: no quality limit, discard short reads = 15, trim 5′ nucleotides = 10, trim 3′ nucleotides = 3, and ambiguous bases = 0. The rRNA sequences for each strain were imported from GenBank, and rRNA was mapped using minor changes to default settings, e.g., the length fraction was adjusted to 0.6, and the unmapped reads were collected for further analysis. A reference genome for each strain was downloaded from GenBank and annotated with Prokka (Seemann, 2014). RNA-Seq analysis was performed on unmapped reads with the selection “genome annotated with genes only” and minor changes to default settings, e.g., length fractions were set to 0.6, and broken pairs were not ignored. Differential gene expression analysis was performed on the data to compare each timepoint to time 0 using Reads Per Kilobase per Million (RPKM) normalization. Fold changes and FDR *p*-values were exported from CLC Genomics Workbench 22, and eggNOG-mapper 2.1.12^1^ was used to assign genes to the appropriate Clusters of Orthologous Groups (COG) category. Bar plots were created using Excel. A quality check was performed by aligning total reads, including rRNA, to the reference genome to check mapping percentage and the results of this can be found in Supplementary Table S1.

### 2.4 qPCR confirmation of RNA-Seq data

RT-qPCR and comparative ΔΔCt analyses were used to confirm the gene expression data from RNA-Seq. The incubation and RNA preparation procedures described in sections 2.1 and 2.2 were repeated to obtain additional RNA, with samples collected in triplicate. RNA samples were analyzed for DNA contamination by conducting PCR with the *23S* gene and analyzing the products by gel electrophoresis. In the aerosensitive *C. jejuni* strain T1-21, five genes (*btuB2*, *cirA*, *clpB*, *hmuU*, and an unknown gene with GenBank locus tag ASB61_RS06375) were selected for RT-PCR since these were expressed at high levels in the RNA-Seq data. In the aerotolerant *C. jejuni* strain WP2202, the genes *clpB*, *dnaK*, *groL*, *groS*, and *hmuU* were analyzed by RT-PCR. The *16S* gene was selected as an internal control based on its frequent usage in the literature and its stable expression in many organisms. A list of primers for the selected genes can be found in Supplementary Table S2.

Reverse transcription was performed using the LunaScript® RT Supermix Kit (NEB #E3010), and qPCR reactions were conducted with the Luna® Universal qPCR Mastermix (NEB #M3003). qPCR reactions were performed in an Applied Biosystems StepOnePlus™ instrument and were executed according to protocols provided with the Luna Universal qPCR kit. Standard curves were prepared for both internal controls to confirm primer efficiency. A no-template RT negative control was created during the reverse transcription step, and this negative control was used with each gene during qPCR. Ct values were obtained from the qPCR data for both target genes and internal controls. Comparative ΔΔCt analysis was performed to compare each gene to the internal control Ct values and to compare each timepoint Ct to time 0. Fold changes were calculated from the ΔΔCt values to determine expression levels. Pearson and Spearman rank correlation coefficients were calculated for RNA-Seq and qPCR fold changes.

## 3 Results

### 3.1 Differences in overall gene regulation

**Figure 1:**
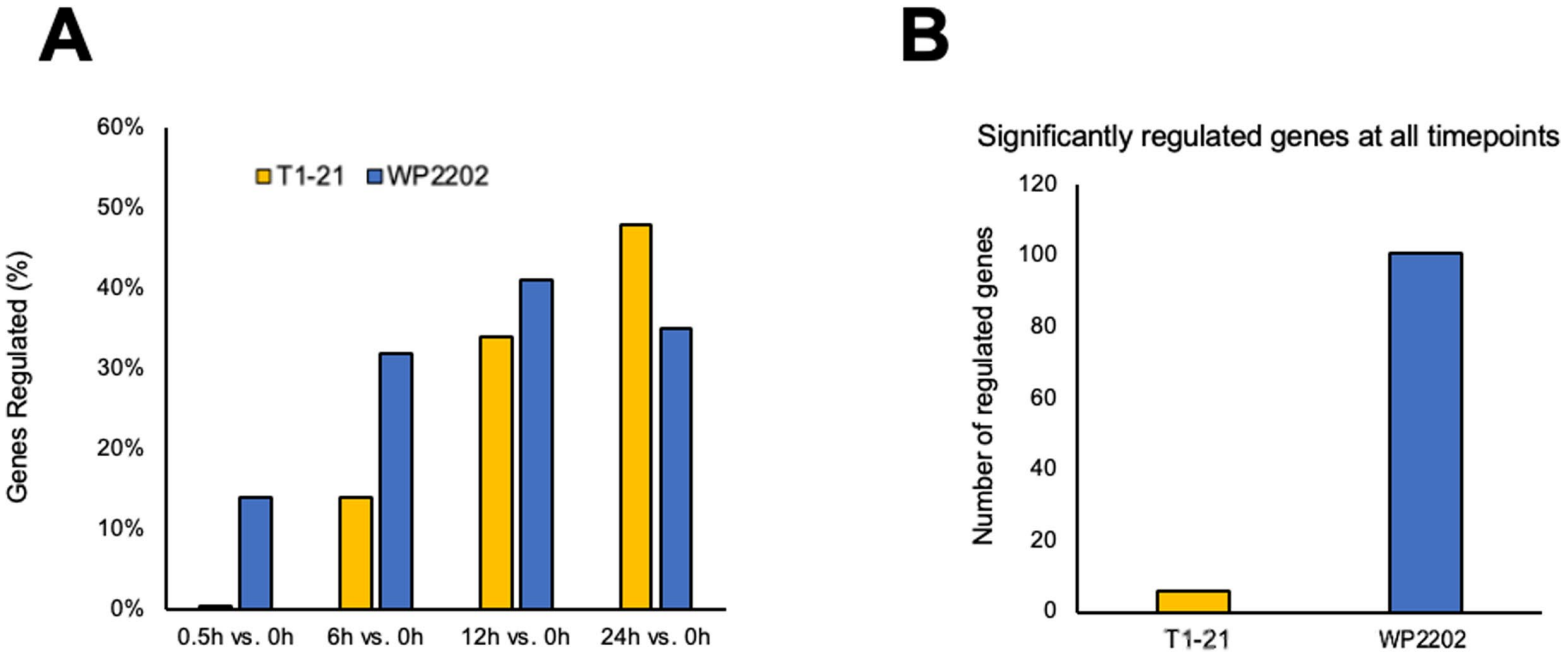
Comparison of gene regulation in the aerosensitive *C. jejuni* strain T1-21 and the aerotolerant strain WP2202, excluding plasmid genes. **(A)** The percentage of genes regulated at each timepoint. **(B)** The number of genes significantly regulated at all timepoints. Significance was defined as FDR *p*-values less than 0.05 and fold-change magnitudes of 1.5 or greater.

Significant differences in gene regulation were observed between the aerotolerant and aerosensitive *C. jejuni* strains. When data was separated by timepoints, the aerotolerant strain WP2202 exhibited a greater number of differentially regulated genes (DEGs) under aerobic conditions as compared to the microaerobic condition than the aerosensitive strain T1-21 at 0.5, 6, and 12 h (Figure 1A). Regulation in the aerotolerant strain peaked at the 12 h timepoint, whereas regulation in the aerosensitive strain peaked at 24 h (Figure 1A). Furthermore, when datapoints were combined, a larger number of genes were differentially regulated in the aerotolerant WP2202 as compared to the aerosensitive T1-21 strain (Figure 1B).

### 3.2 Regulatory changes in different COG categories

**Figure 2:**
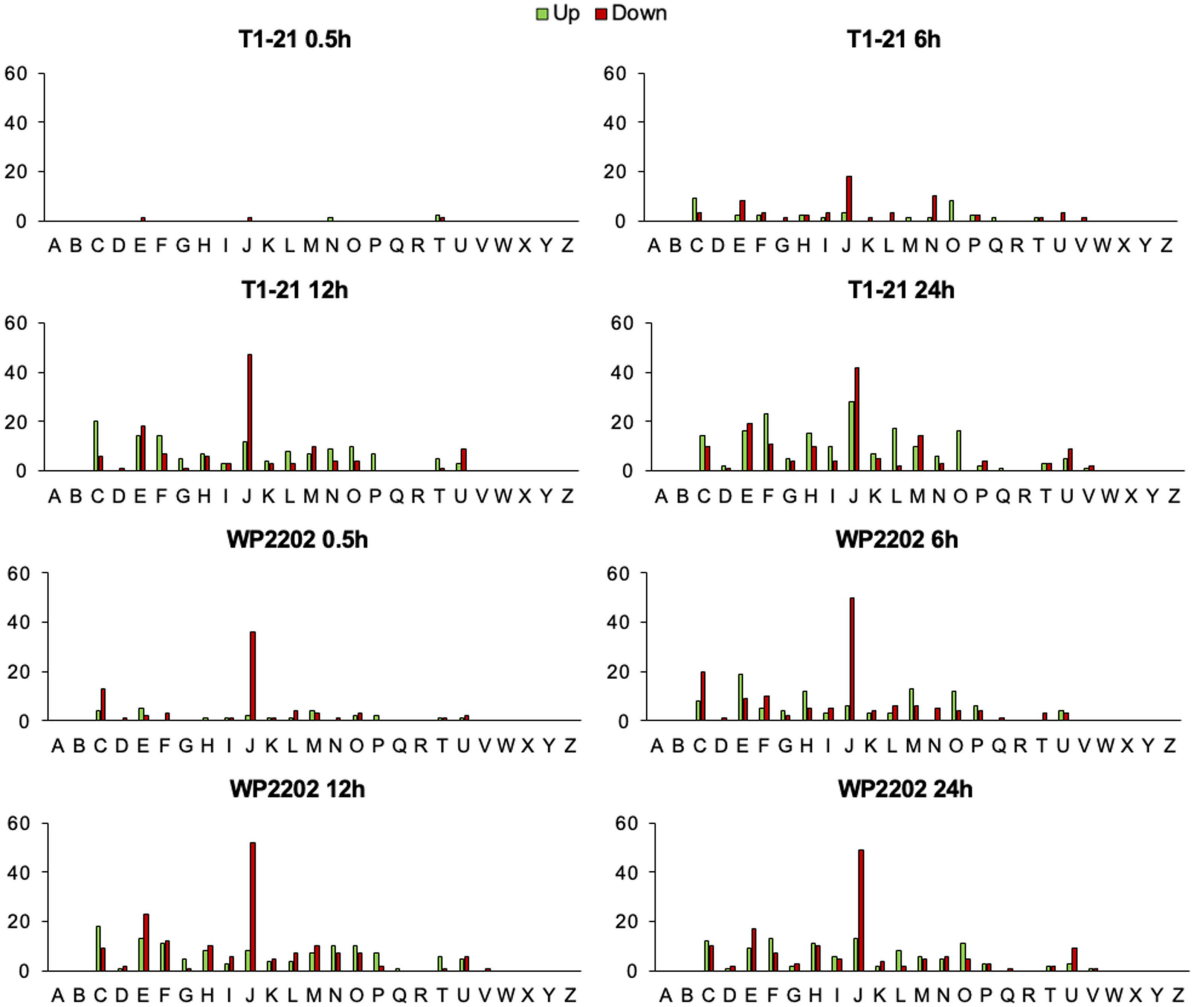
Number of genes significantly up- or down-regulated in different COG categories. Plasmid genes are excluded. Datapoints represent gene regulation in the aerosensitive *C. jejuni* T1-21 and aerotolerant WP2202 as a function of time. COG Category S was removed to allow for more effective scaling of *y*-axes. Significance was defined as FDR *p*-values less than 0.05 and fold-change magnitudes of 1.5 or greater. (A) RNA processing and modification. (B) Chromatin structure and dynamics. (C) Energy production and conversion. (D) Cell cycle control, cell division, and chromosome partitioning. (E) Amino acid transport and metabolism. (F) Nucleotide transport and metabolism. (G) Carbohydrate transport and metabolism. (H) Coenzyme transport and metabolism. (I) Lipid transport and metabolism. (J) Translation, ribosomal structure, and biogenesis. (K) Transcription. (L) Replication, recombination, and repair. (M) Cell wall/membrane/envelope biogenesis. (N) Cell motility. (O) Posttranslational modification, protein turnover, chaperones. (P) Inorganic ion transport and metabolism. (Q) Secondary metabolites biosynthesis, transport and catabolism. (R) General function prediction only. (T) Signal transduction mechanisms. (U) Intracellular trafficking, secretion, and vesicular transport. (V) Defense mechanisms. (W) Extracellular structures. (X) Mobilome: prophages, transposons. (Y) Nuclear structure. (Z) Cytoskeleton.

The gene expression data was sorted into COG categories to analyze trends in regulation. The COG category with the highest number of DEGs under aerobic conditions across all timepoints and both strains was COG category S, which includes genes with unknown functions. COG category S was not included in Figure 2 to allow for more effective scaling of the *y*-axis; however, a version of this figure that includes COG category S can be found in Supplementary Figure S1. Genes were only regarded as differentially regulated if they had FDR *p*-values below 0.05 and fold changes with a magnitude of 1.5 or greater.

When category S was excluded, COG category J (translation, ribosomal structure, and biogenesis) had the highest number of DEGs with downregulated genes occurring at most timepoints in the two strains (Figure 2), peaking at 70 genes up- or down-regulated at the 24-h timepoint of the aerosensitive strain. COG category C (energy production and conversion) was generally upregulated in the aerosensitive T1-21 strain. In the aerotolerant WP2202 strain, COG category C was downregulated at 0.5 and 6 h and upregulated at 12 and 24 h. In the aerosensitive T1-21 strain, genes in COG Category E (amino acid transport and metabolism) were downregulated at 6 h followed by similar numbers of up- and down-regulated genes at 12 h and 24 h. In the aerotolerant WP2202, genes in COG category E were upregulated at 6 h and downregulated at 12 and 24 h. DEGs in COG category F (nucleotide transport and metabolism) were upregulated in the aerosensitive T1-21 strain at 12 and 24 h. In the aerotolerant WP2202, DEGs in COG category F were downregulated at 6 h and upregulated at 24 h.

### 3.3 Analysis of genes associated with aerotolerance

**Figure 3:**
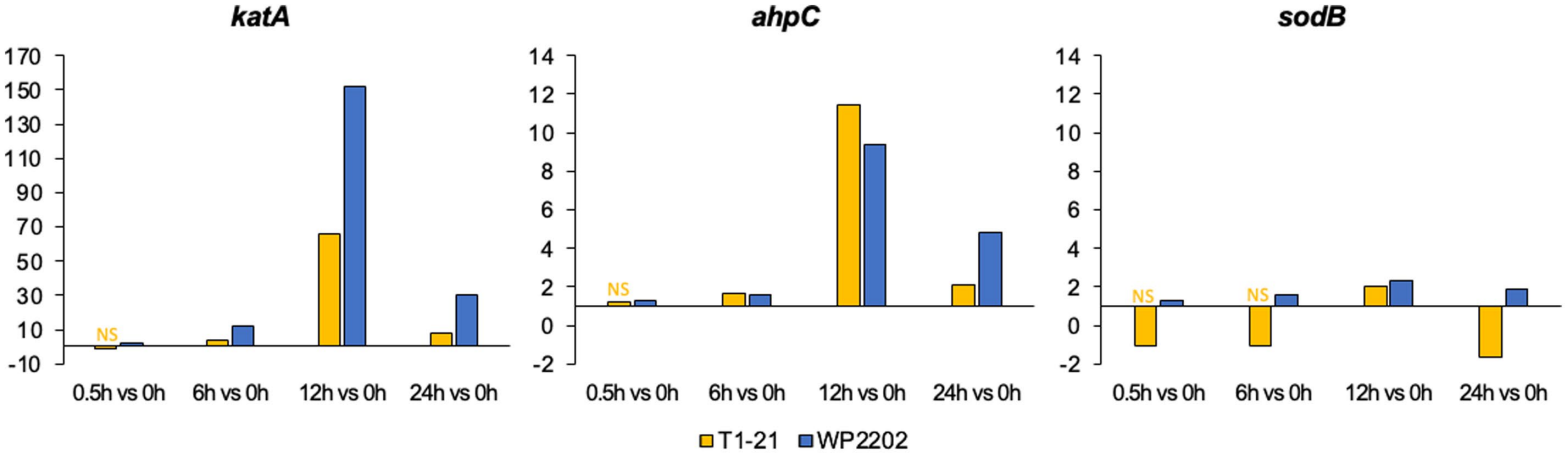
Fold changes in *ahpC*, *sodB*, and *katA* expression in in the aerosensitive *C. jejuni* strain T1-21 and the aerotolerant strain WP2202. “NS” indicates nonsignificant data. Significance is defined as an FDR *p*-value less than 0.05. The *y*-axes are scaled differently on each graph due to variability in fold-change magnitudes.

As mentioned in the introduction, *katA*, *ahpC*, and *sodB* have been investigated in *Campylobacter* for their role in aerotolerance (Vercellone et al., 1990; Purdy and Park, 1994; Baillon et al., 1999). In a closer analysis of the RNA-Seq data, *katA* expression was strongly induced at the 12-h timepoint in both strains and was 66-fold and 152-fold higher in the aerosensitive T1-21 and aerotolerant WP2202 strains, respectively, when compared to expression at 0 h (Figure 3). Furthermore, *katA* expression at 24 h remained 30-fold higher than levels at the 0 h timepoint. Expression of *ahpC* was also upregulated at 12 h and was 11 and 9-fold higher in the aerosensitive and aerotolerant strains, respectively (Figure 3). With respect to *sodB*, expression was nonsignificant at 0.5 or 6 h in the aerosensitive strain T1-21; however, *sodB* transcript levels underwent a two-fold increase as compared to the microaerobic condition at 12 h, which was followed by a decease in expression at 24 h (Figure 3). Mild upregulation of *sodB* was observed at all timepoints in the aerotolerant strain, with fold changes ranging from 1.3 to 2.3 (Figure 3).

**Figure 4:**
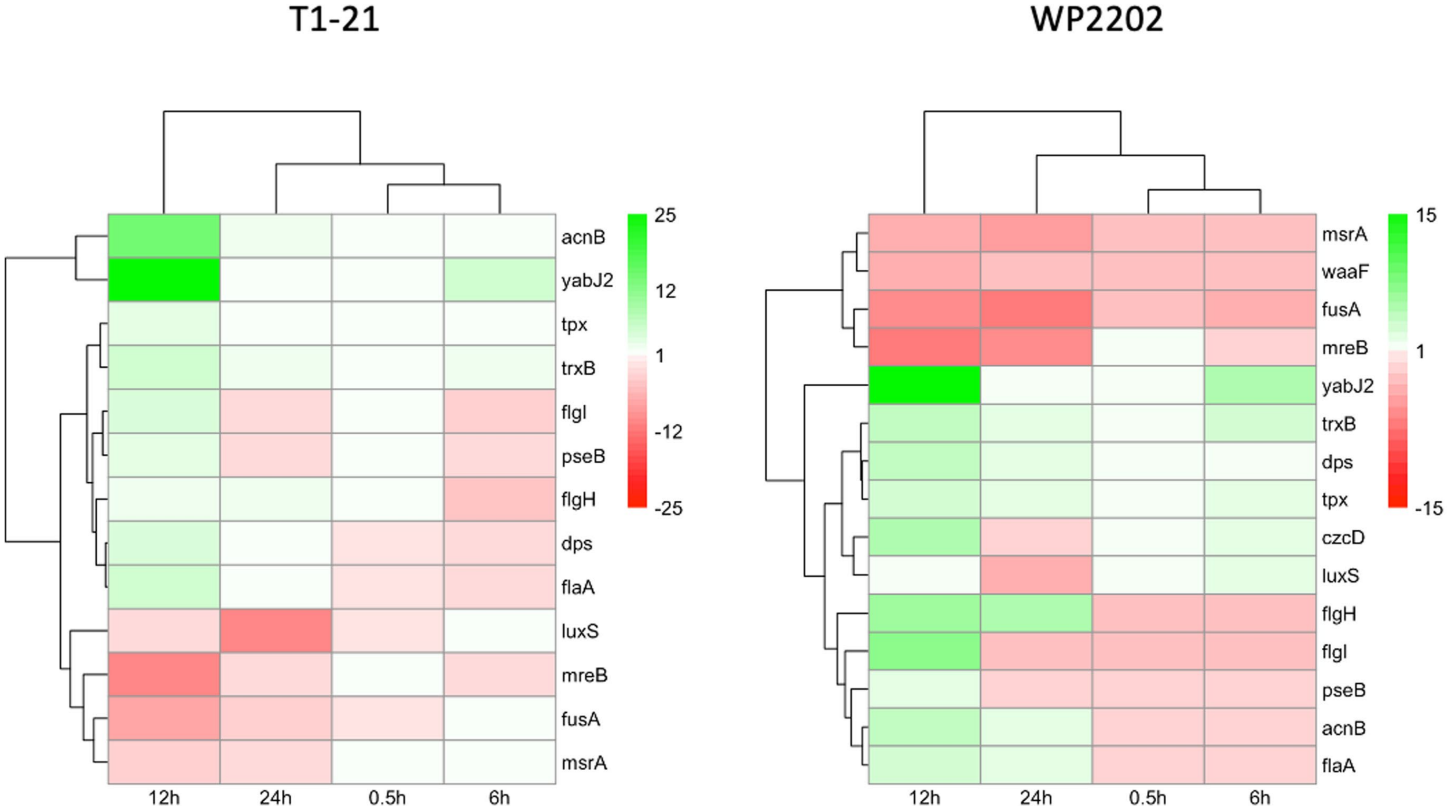
Heatmap of fold changes at each timepoint for genes studied previously in the literature for *C. jejuni* aerosensitive strain T1-21 and aerotolerant strain WP2202. Genes were selected based on prior studies suggesting their involvement in aerotolerance and/or the oxidative stress response. Red indicates downregulation and green indicates upregulation. Nonsignificant timepoints are included. Average of fragment fold changes were taken for each timepoint of the WP2202 *flaA* fragments (*flaA_1*, *flaA_2*, *flaA_3*, and *flaA_4*).

Other *Campylobacter* genes reported to function in aerotolerance include *acnB*, *czcD*, *dps*, *flaA*, *flgI*, *flgH*, *fusA*, *luxS*, *mreB*, *msrA*, *pseB*, *tpx*, *trxB*, and *waaF* (Ishikawa et al., 2003; Atack and Kelly, 2008; Atack et al., 2008; Garénaux et al., 2008; He et al., 2008; Hwang et al., 2011; Flint et al., 2014; Handley et al., 2015; Rodrigues et al., 2016; Kim et al., 2023; Stoakes et al., 2024). Additionally, the gene *yabJ2* is noted for its relation to capsule formation since capsules have been suggested as a mechanism behind aerotolerance (Kim et al., 2023). Varying levels of *acnB*, *tpx*, and *trxB* upregulation were observed at all significant timepoints (FDR *p*-values < 0.05) in both strains (Figure 4). Interestingly, *czcD* was upregulated at 0.5, 6, and 12 h in the aerotolerant WP2202, but was not present in the data for the aerosensitive T1-21 (Figure 4). The genes *fusA* and *mreB* were downregulated at all significant timepoints in both strains (Figure 4). The *msrA* gene was downregulated in the aerotolerant strain, but all timepoints in the aerosensitive were nonsignificant (Figure 4). Similarly, *waaF/rfaF* was downregulated in the aerotolerant WP2202 strain but was not found in the aerosensitive T1-21 (Figure 4). The two strains exhibited variable expression for *dps*, *flaA*, *flgI*, *flgH*, *luxS*, and *pseB* (Figure 4). Additionally, *yabJ2* was highly upregulated (fold change > 10) at the 12-h timepoint in both strains (Figure 4).

**Figure 5:**
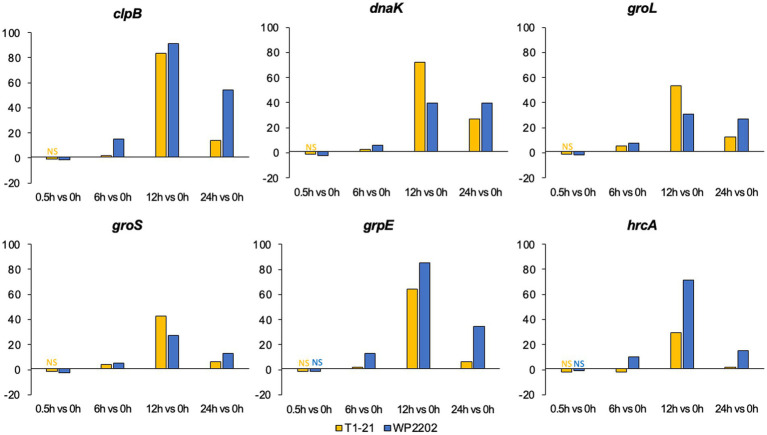
Fold changes in the heat shock response genes *clpB*, *dnaK*, *groL*, *groS*, *grpE*, and *hrcA* in the aerosensitive *C. jejuni* strain T1-21 and the aerotolerant strain WP2202. “NS” indicates nonsignificant data. Significance is defined as an FDR *p*-value less than 0.05.

A number of genes with roles in the heat shock response were upregulated in the RNA-Seq data, including *clpB*, *dnaK*, *groL*, *groS*, *grpE*, and *hrcA* (Figure 5) (Squires et al., 1991; Thies et al., 1999a, 1999c; Parkhill et al., 2000; Andersen et al., 2005; Holmes et al., 2010; Sung et al., 2024). Previous studies have suggested that heat shock genes may play a role in aerotolerance (Andersen et al., 2005; Brøndsted et al., 2005; Boehm et al., 2015). Expression patterns were similar for all six genes in the aerotolerant WP2202 strain; these genes were initially downregulated at 0.5 h and then upregulated at all other timepoints (Figure 5). A similar expression pattern was observed for these genes in the aerosensitive T1-21 strain. Both strains exhibited strong upregulation of the six genes at 12 h (Figure 5). It is important to mention that expression at the 0.5 h timepoint was insignificant for these six genes in the aerosensitive strain and for *grpE* and *hrcA* in the aerotolerant strain (Figure 5).

**Figure 6:**
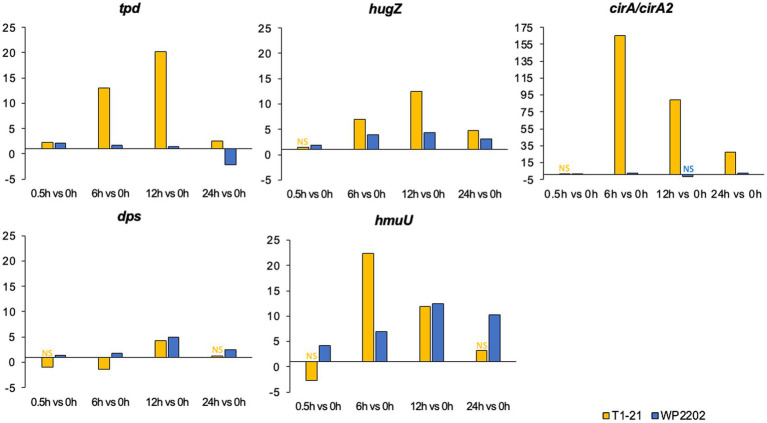
Fold changes in the expression of iron regulatory genes *tpd*, *hugZ*, *cirA2*, *hmuU*, and *dps* in the aerosensitive *C. jejuni* strain T1-21 and the aerotolerant strain WP2202. “NS” indicates nonsignificant data. Significance is defined as an FDR *p*-value less than 0.05. The *y*-axes are scaled differently to account for differences in fold-change magnitude.

*Campylobacter* genes *tpd*, *hugZ*, *cirA2*, *hmuU*, and *dps* have potential roles in iron acquisition (Griggs et al., 1987; Ishikawa et al., 2003; Ridley et al., 2006; Cuív et al., 2008; Chan et al., 2010; Rodrigues et al., 2016; Müller et al., 2020). Iron acquisition genes have previously been noted as possibly playing a role in aerotolerance (Wai et al., 1996; van Vliet et al., 2001, 2002; Ishikawa et al., 2003; Garénaux et al., 2008; Flint et al., 2014; Rodrigues et al., 2016). Interestingly, *tpd* was upregulated in the aerosensitive T1-21 at all timepoints, with notable increases at 6 h (fold change = 13) and 12 h (fold change = 20) (Figure 6). In the aerotolerant WP2202, *tpd* was slightly upregulated (fold change 1.4–2.1) at 0.5 to 12 h, but was downregulated at 24 h (fold change −2.1) (Figure 6). In both strains, *hugZ* was upregulated at all timepoints with higher expression in the aerosensitive T1-21 strain (fold changes ranging from 4.8 to 12) vs. the aerotolerant WP2202 (fold changes ranging from 1.9 to 4.3) (Figure 6). A large increase in *cirA2* expression was observed in the aerosensitive T1-21 beginning at 6 h (fold change 165) and continued to be elevated at 24 h (Figure 6). Interestingly, *cirA2* was not annotated by Prokka in the data for WP2202; however, the *cirA* allele in the aerotolerant WP2202 was 93% identical to *cirA2* in T1-21. In the WP2202 data, *cirA* was slightly upregulated at 0.5, 6, and 24 h but was nonsignificant data at 12 h. In both strains, expression of *hmuU* was generally upregulated and was most highly expressed at 6 h (fold change 22) in the aerosensitive T1-21 (Figure 6). Expression of *dps* in T1-21 was slightly downregulated at 6 h and mildly upregulated at 12 h; expression at 0.5 and 24 h was nonsignificant (Figure 6). In WP2202, *dps* was upregulated at all timepoints (Figure 6).

**Figure 7:**
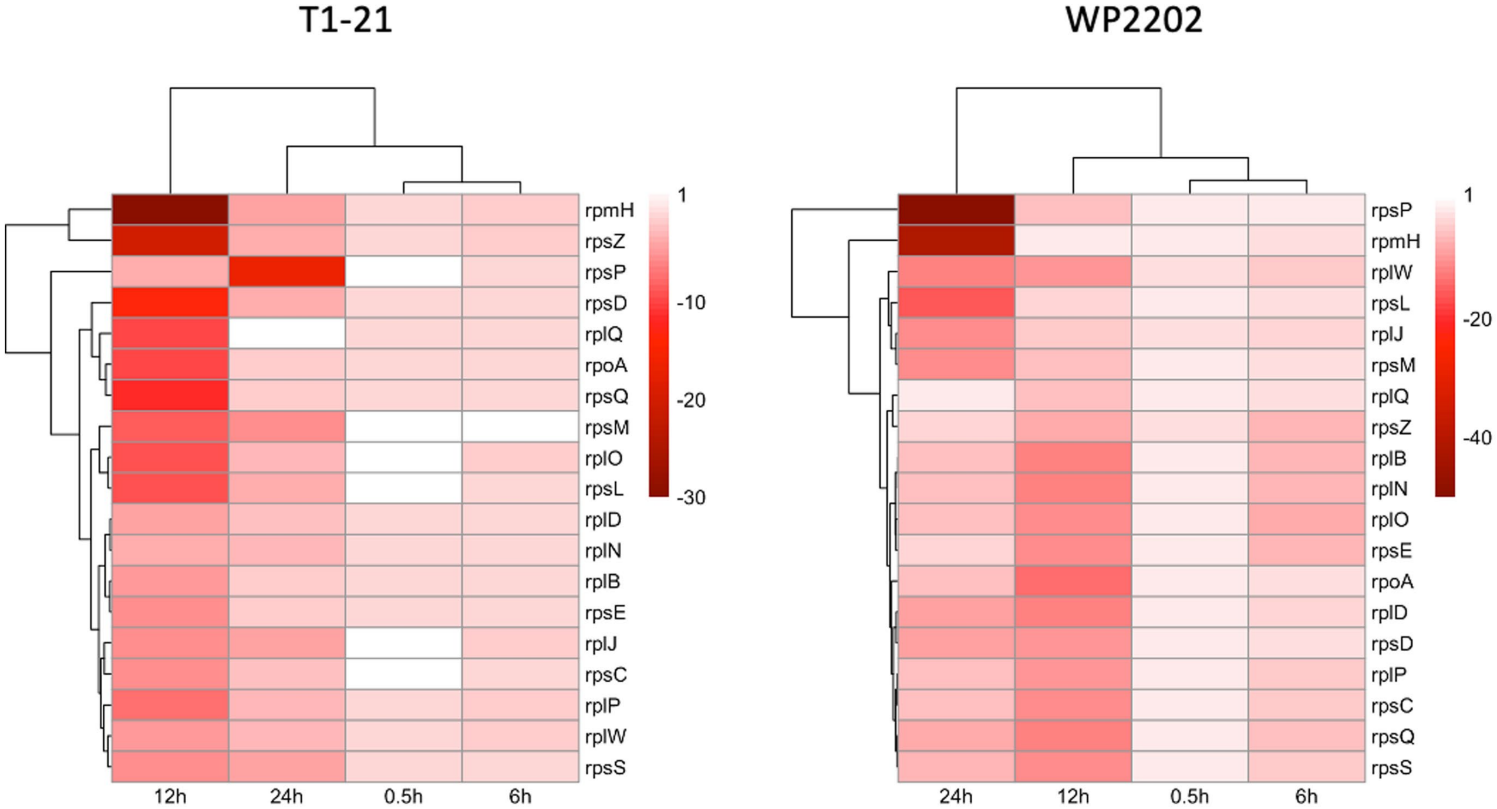
Heatmap of fold changes in expression at each timepoint for 18 ribosomal genes in the aerosensitive *C. jejuni* strain T1-21 and the aerotolerant strain WP2202. Darker red represents a greater magnitude of downregulation. Nonsignificant timepoints are included.

RNA-Seq analysis revealed multiple ribosomal genes that had notable differences in expression, including *rpmH*, *rpsZ*, *rplB*, *rplD*, *rplJ*, *rplN*, *rplO*, *rplP*, *rplQ*, *rpsC*, *rpsD*, *rpsE*, *rpsL*, *rpsM*, *rpsP*, *rpsQ*, *rpsS*, *rplW*, and *rpoA*. These genes were downregulated at all timepoints in both strains (Figure 7). The majority of these genes were more highly downregulated in the aerotolerant WP2202 as compared to the aerosensitive T1-21; an exception was *rpsZ*, where the pattern was reversed.

**Figure 8:**
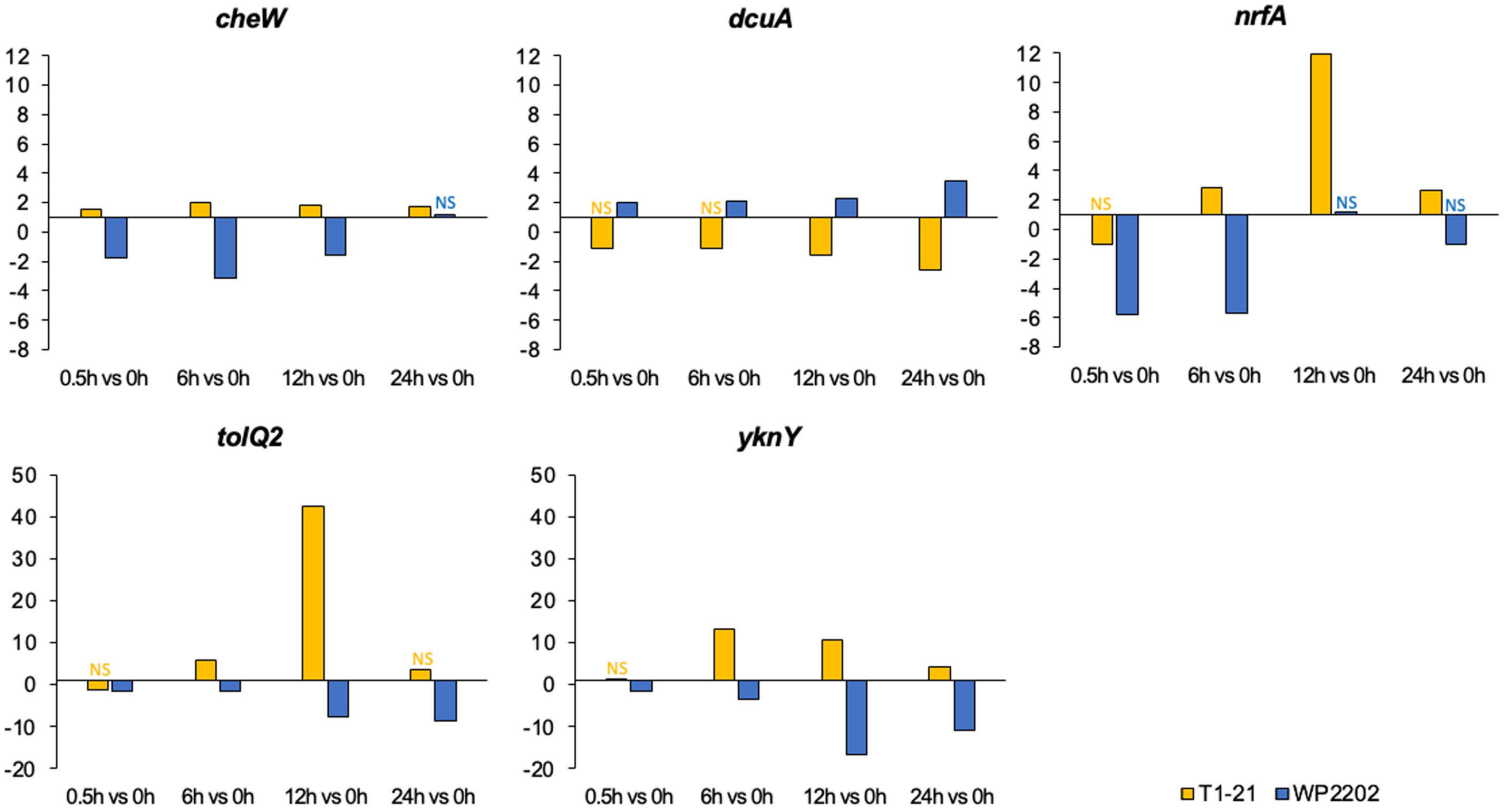
Fold changes of genes that were up- or down-regulated in the *C. jejuni* aerosensitive strain T1-21 but not the aerotolerant strain WP2202. “NS” indicates nonsignificant data. Significance was defined as an FDR *p*-value less than 0.05. The *y*-axes were scaled differently on some of the graphs due to differences in fold-change magnitudes.

Interestingly, *cheW*, *dcuA*, *nrfA*, *tolQ2*, and *yknY* genes were upregulated in one but not the other strain. For example, *cheW*, *nrfA*, and *yknY* were upregulated at all significant timepoints in the aerosensitive T1-21 strain but were downregulated in the aerotolerant WP2202 strain (Figure 8). In the aerosensitive T1-21 strain, *tolQ2* was upregulated at 6 and 12 h (fold change 42 at 12 h), but was downregulated in the aerotolerant WP2202 strain (Figure 8). In contrast, *dcuA* was upregulated at all timepoints in the aerotolerant WP2202 but was downregulated in the aerosensitive T1-21 strain (Figure 8).

We hypothesized that genes highly expressed at 0.5 and 6 h may be recruited as part of a fast response to aerobic stress. Genes that were highly up- or down-regulated (fold change magnitudes > 10) at 0.5 or 6 h in the aerosensitive T1-21 included *btuB1*, *btuB2*, *cirA2*, *btuF*, *hmuU*, *tpd*, and *yknY*, and three unknown genes (GenBank locus tags ASB61_RS03560, ASB61_RS08465 on plasmid pcjDM, and a region overlapping locus tags ASB61_RS07715, ASB61_RS07720, and ASB61_RS07725) (Supplementary Tables S3, S4). A gene annotated in Prokka as unknown mapped to a region overlapping *chuA* and *chuB* was also highly upregulated at 0.5 and 6 h. In the aerotolerant WP2202, *clpB*, *grpE*, *hrcA*, *katA*, *kdgR* and four unknown genes (GenBank locus tags A0W69_06090, A0W69_06100, A0W69_08085, and a region overlapping locus tags A0W69_08115 and A0W69_08120) were highly upregulated at 0.5 and 6 h (Supplementary Table S5). Two unknown genes (GenBank locus tags A0W69_01600 and a region overlapping locus tags A0W69_08545 and A0W69_08550) were highly downregulated in the aerotolerant WP2202 strain.

Numerous genes showed large increases or decreases in expression (fold-change magnitudes > 10) at 12 or 24 h. Genes that showed changes in expression in both strains included *cfa*, *dnaK*, *fabD*, *glnA*, *gltB1*, *groL*, *groS*, *grpE*, *hmuU*, *hrcA*, *katA*, *nrgA*, *psd1*, *secY*, *trpGD*, *wbpE*, *yabJ2,* and *yidC* (Supplementary Tables S3, S5). Genes that showed altered expression in the aerosensitive T1-21 but not the aerotolerant WP2202 included *acnB*, *ahpC*, *clpB*, *flaC*, *hugZ*, *mdtC*, *nrfA*, *pfs*, *purL*, *secD*, *tolQ2, trpB, ttca,* and seven unknown genes on plasmid pcjDM (locus tags ASB61_RS08740, ASB61_RS08640, ASB61_RS08525, ASB61_RS08545, ASB61_RS08600, ASB61_RS08440, and ASB61_RS08430) (Supplementary Tables S3, S4). In the aerotolerant strain, genes with large changes in expression included *atpC*, *dedA*, *ligA2*, *mmpA*, *modA*, *pth*, *purH*, *tal*, *tktA*, *torC*, and *torZ* (Supplementary Table S5).

### 3.4 Confirmation of RNA-Seq results by qPCR

**Figure 9:**
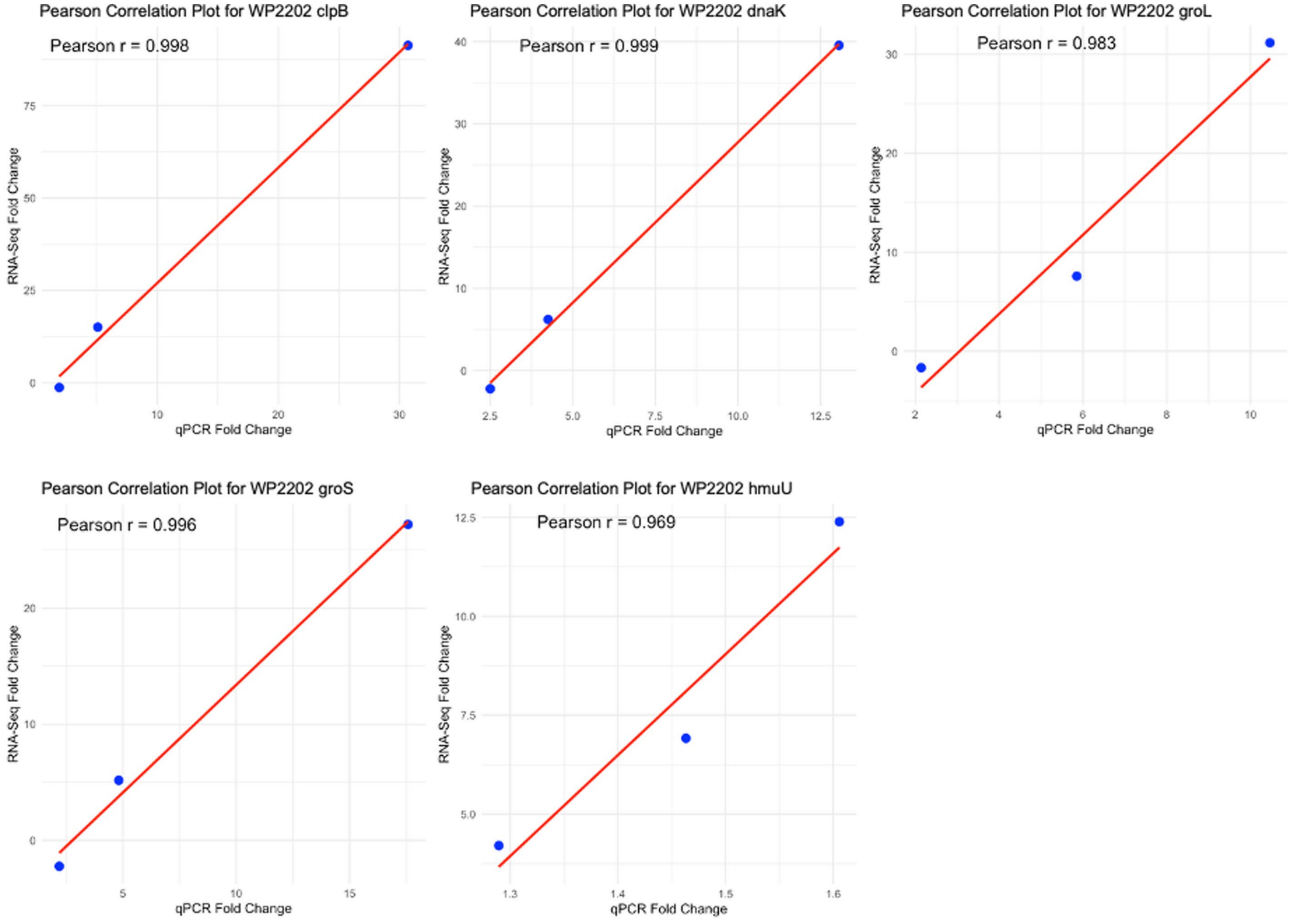
Confirmation of RNA-Seq results by qPCR. The five scatterplots show Pearson correlation plots for *clpB*, *dnaK*, *groL*, *groS*, and *hmuU* in the aerotolerant strain WP2202.

The reliability of the RNA-Seq data obtained for the aerotolerant WP2202 strain was evaluated at 0.5, 6 and 12 h. The Spearman (data not shown) and Pearson correlation coefficients (Figure 9) showed strong positive correlations for the five genes analyzed (*clpB*, *dnaK*, *groL*, *groS*, and *hmuU*) in WP2202.

Issues were encountered during the statistical analysis of qPCR data for the aerosensitive T1-21 due to the limited number of data points for evaluating the correlation between RNA-Seq and qPCR data. Many genes in the aerosensitive T1-21 strain were statistically nonsignificant at the 0.5 h timepoint in the RNA-Seq data, so this time was not included in the analysis. Additionally, low amounts of RNA were obtained at the 24-h timepoint for both strains during the qPCR analysis, so this timepoint was also excluded. The remaining timepoints (6 and 12 h) were positively correlated via the Spearman correlation coefficient analysis for *btuB2*, *cirA*, *clpB*, and the unknown gene with GenBank locus tag ASB61_RS06375; however, a negative correlation was observed for *hmuU*. Analysis using the Pearson correlation coefficient was not possible with only two timepoints.

## 4 Discussion

In this study, we compare differences in gene expression in the aerosensitive T1-21 and aerotolerant WP2202 strains of *C. jejuni* during microaerobic conditions (time 0) and aerobic conditions (times 0.5, 6, 12, and 24 h). The goal of comparing gene expression in the aerotolerant and aerosensitive strains was to identify potential genetic mechanisms that allow the aerotolerant strain to survive the same level of O_2_ exposure that is lethal to the aerosensitive strain. Additionally, we expected that gene expression in the two strains would change in aerobic conditions as the bacterial cells attempt to survive O_2_ exposure, which is why the comparison of gene expression in aerobic and microaerobic conditions is important. Identifying the genetic mechanisms underlying aerotolerance in *C. jejuni* and its response to oxidative stress is vital for understanding the ability of this bacterium to survive O_2_ exposure during meat processing and for developing possible strategies to reduce the contamination of our food supply with this pathogen.

Clear differences in gene regulation were found in the two strains of *C. jejuni*. Interestingly, the aerotolerant WP2202 differentially regulated a greater percentage of genes under aerobic conditions than the aerosensitive T1-21 strain at 0.5, 6, and 12 h but not at 24 h. It is possible that the T1-21 cells begin to die at 24 h due to prolonged exposure to oxidative stress, and the uptick in gene expression at this timepoint may be a final attempt at survival. Although prolonged exposure to oxidative stress at 24 h would likely be lethal to the aerosensitive T1-21 strain, the continued changes in gene expression suggest that some of the bacterial cells were surviving. At 24 h, the T1-21 strain may be entering the viable-but-nonculturable (VBNC) state, which is a strategy used by *C. jejuni* to cope with oxidative stress (Moran and Upton, 1987a; Lee et al., 2005; Zhong et al., 2020). One study indicates *cj1254* as potentially vital for the VBNC state (Ohno et al., 2024), and a close homolog to the gene (locus tag LBCKBIAG_01152) was found to be upregulated (fold change of 2.7) at 24 h in the aerosensitive strain in our study, however its regulation was nonsignificant at the other timepoints (Supplementary Table S3). In contrast, gene expression in the aerotolerant WP2202 strain began to increase earlier than in the aerosensitive T1-21, with an initial spike in regulation starting at 0.5 h that continued to increase up to 12 h. The rapid change in gene expression exhibited by WP2202 in response to aerobic stress likely improves its ability to survive O_2_ exposure for longer durations than the aerosensitive T1-21 strain. Additionally, WP2202 exhibited widespread downregulation of genes in its plasmid, possibly as a strategy to conserve resources under oxidative stress (Supplementary Table S6).

The number of genes significantly regulated in each COG category was recorded for the purpose of noting general trends in regulation that could indicate what pathways are differentially regulated in response to aerobic conditions. The majority of genes were assigned to COG category S, which includes all genes of unknown function; this data reveals that much of the *C. jejuni* genome remains uncharacterized. The category with the second largest number of genes differentially regulated was COG category J (translation, ribosomal structure, and biogenesis). In the aerotolerant WP2202, a large number of genes in COG category J were downregulated at all timepoints, possibly because the strain was conserving resources during aerobic stress. Unlike the WP2202 strain, the T1-21 strain upregulated several category J genes at 24 h, perhaps as part of a last-minute survival attempt. The other COG categories represented in the RNA-Seq data contained fewer DEGs than those in category J. In the aerosensitive T1-21, the upregulation of genes in COG category C (energy production and conversion) may have been to produce the additional energy needed to fuel the oxidative stress response. In the aerotolerant WP2202 strain, category C genes were primarily downregulated at 0.5 and 6 h, followed by upregulation at 12 and 24 h. If the aerotolerant strain was coping well with oxidative stress, upregulation of category C genes may have been unnecessary until prolonged oxidative stress occurred. The aerotolerant WP2202 upregulated COG category E (amino acid transport and metabolism) genes at 6 h, followed by downregulation at 12 and 24 h. The early upregulation of category E genes in aerotolerant WP2202 could be part of the strain’s stress response, whereas downregulation at 12 h and 24 h may reflect a need to save resources after prolonged stress. Both strains apparently benefit from upregulating genes from COG category F (nucleotide transport and metabolism) at the later timepoints, suggesting that nucleotide transport and metabolism is important after prolonged stress. Genes in COG category H (coenzyme transport and metabolism) were upregulated in the aerotolerant WP2202 at 6 h, perhaps as part of the oxidative stress response. Differential regulation of category H genes in the aerosensitive T1-21 was not obvious until 12 h. Based on these differences in the two strains, it is plausible that the early upregulation of category H genes in the aerotolerant strain is important for O_2_ tolerance. Coenzymes are known to have roles in the oxidative stress response in bacteria (Gout, 2019), which suggests that this category of genes might help the organism survive O_2_ exposure.

The *katA*, *ahpC*, and *sodB* genes have been well-studied for their roles in the oxidative stress response in *Campylobacter* (Vercellone et al., 1990; Purdy and Park, 1994; Baillon et al., 1999). Our data show that these three genes were differentially regulated in both the aerosensitive T1-21 and the aerotolerant WP2202. Expression of *katA* gene, which encodes a catalase that helps protect bacteria from hydrogen peroxide (Verhoeff-Bakkenes et al., 2008), was highly upregulated (fold change > 60) in both strains at 12 h and expression was over two-fold higher in the WP2202 as compared to T1-21. Furthermore, *katA* expression was higher in the aerotolerant WP2202 strain versus T1-21 at 6 and 24 h, which likely contributes to the higher aerobic fitness of the aerotolerant strain. With respect to *katA*, our results agree with other studies showing increased levels of catalase activity in *Campylobacter* spp. during oxidative stress, including studies conducted with aerotolerant strains (Vercellone et al., 1990; Rodrigues et al., 2016; Oh et al., 2019).

The *ahpC* gene encodes an alkyl hydroperoxide reductase that protects DNA from oxidative stress (Jacobson et al., 1989). Similar to *katA*, an uptick in *ahpC* expression was observed at 12 h for both WP2202 and T1-21, with a slightly higher magnitude (11-fold change) in the aerotolerant strain. This data provides further evidence that the oxidative stress response is particularly important at the 12 h timepoint in both strains. Our data show a similar level of *ahpC* upregulation in both T1-21 and WP2202, which differs from another study where this gene was more highly regulated in an aerotolerant strain of *C. jejuni* as compared to an aerosensitive reference strain (Rodrigues et al., 2016).

Superoxide dismutase, which is encoded by *sodB*, is involved in the detoxification of reactive oxygen species (ROS) (Fridovich, 1983; Purdy and Park, 1994). Mild upregulation of *sodB* was observed at all timepoints in the aerotolerant WP2202 strain, suggesting a role in the oxidative stress response. In the aerosensitive strain T1-21, upregulation of *sodB* was observed only at the 12-h timepoint and was downregulated at 24 h, with the other timepoints nonsignificant. Our data for *sodB* agrees with prior studies that indicate its role in aerotolerance (Kikuchi and Suzuki, 1984; Lee et al., 2005; Rodrigues et al., 2016; Oh et al., 2019).

Numerous other genes were noted in the data based on previous studies showing a relationship with aerotolerance or the oxidative stress response. Three genes, *acnB*, *tpx*, and *trxB*, were upregulated at statistically significant timepoints in both strains, which agrees with studies showing a potential role for these genes in the oxidative stress response (Atack et al., 2008; Flint et al., 2014; Rodrigues et al., 2016). Another gene, *czcD*, was not found in the RNA-Seq data for the aerosensitive T1-21 strain but was upregulated at all statistically significant timepoints in the aerotolerant WP2202. A prior study suggested a role for *czcD* in resistance to H_2_O_2_ (Stoakes et al., 2024), so it remains possible that *czcD* contributes to aerotolerance in WP2202. CzcD is also involved in zinc export, providing protection against toxic zinc levels potentially imposed by the host as an antimicrobial strategy (Barnawi et al., 2020). Although *fusA* and *mreB* were previously shown to be induced by paraquat, which causes oxidative stress (Garénaux et al., 2008), these two genes were downregulated in our RNA-Seq data, indicating they may not have roles in T1-21 or WP2202 aerotolerance. A previous study indicated that a *msrA* mutant exhibited decreased resistance to oxidative stress (Atack and Kelly, 2008). In the aerotolerant WP2202, *msrA* was downregulated at 12 and 24 h; however, *msrA* expression at other timepoints in WP2202 and all timepoints in T1-21 were statistically nonsignificant. The *waaF/rfaF* gene is known to be involved in capsule formation, and a *waaF* mutant had decreased aerotolerance (Kim et al., 2023). Although *waaF* was not present in the aerosensitive T1-21 strain, it was downregulated in the aerotolerant WP2202 strain. In *S. aureus*, *yabJ2* was reported to have a potential role in capsule formation (Meier et al., 2007). In the RNA-Seq data for both T1-21 and WP2202, *yabJ2* was upregulated at all statistically significant timepoints with an uptick in expression at 12 h. In a previous study, *dps* was upregulated in *C. jejuni* under aerobic conditions (Rodrigues et al., 2016) and may function to protect the species from oxidative stress by sequestering iron (Ishikawa et al., 2003). In WP2202, *dps* was upregulated at all timepoints, which suggests a role in aerotolerance. The flagellin gene *flaA* was shown to be induced during exposure of *C. jejuni* to paraquat (Garénaux et al., 2008). In our RNA-Seq data, *flaA* was induced in both T1-21 and WP2202 at 12 and 24 h, suggesting this gene may function after prolonged exposure to oxidative stress. In *C. jejuni*, *flgI*, *pseB,* and *flgH* function in motility, and mutations in these genes render the bacterium more susceptible to oxidants (Flint et al., 2014). Oddly, our data showed that *flgI* was downregulated at all significant timepoints in both strains except 12 h where it was slightly upregulated. Similarly, *pseB* was slightly upregulated at the 12 h timepoint in T1-21 but was downregulated at all other significant timepoints. Most of the RNA-Seq data for *pseB* expression in strain WP2202 was not significant. Furthermore, *flgH* showed a similar pattern of initial downregulation followed by upregulation at the 12 and 24 h timepoints in both strains. It remains plausible that *C. jejuni* is upregulating motility genes in an attempt to escape oxidative stress. When the quorum-sensing gene *luxS* was mutated in *C. jejuni*, the bacterium showed increased sensitivity to certain oxidants (He et al., 2008). Our data for both strains showed that *luxS* was mildly upregulated at 6 h and downregulated at 24 h, suggesting that it could have an early function in the oxidative stress response.

Our RNA-Seq data revealed additional genes that might have dual roles in responding to both heat and oxidative stress. The *clpB*, *dnaK, grpE,* and *hrcA* gene products function as proteins or regulators of the heat shock response (Thies et al., 1999a, 1999b; Andersen et al., 2005; Ayllón et al., 2017), and *groS* and *groL* are known to be upregulated during heat stress in *E. coli* (Chueca et al., 2017). Our data for both T1-21 and WP2202 showed that *clpB*, *dnaK*, *groL*, *groS*, and *grpE* were upregulated at 6, 12 and 24 h. The expression pattern in the aerosensitive T1-21 strain was similar for *hrcA* except upregulation occurred later at 12 and 24 h. In both *C. jejuni* strains, these genes exhibited an uptick in upregulation at 12 h, which supports the dual function of these genes in response to both the heat and oxidative stress.

Iron is generally viewed as a causal agent in oxidative stress due to its role in the formation of ROS (Touati, 2000). Interestingly, the addition of iron to growth media was shown to increase aerotolerance in *Campylobacter* (Bowdre et al., 1976; Bolton and Coates, 1983; Moran and Upton, 1987b; Tangwatcharin et al., 2007) and several genes involved in iron acquisition function in aerotolerance, including *dps*, *cft*, and *tonB2* (Wai et al., 1996; Ishikawa et al., 2003; Flint et al., 2014). In our RNA-Seq data, *tpd* (encoding P19)*, hugZ* (Cj1613c), and *hmuU* were highly upregulated in the aerosensitive T1-21 strain but only slightly induced in the aerotolerant WP2202. These genes have presumptive roles in iron acquisition and regulation (Ridley et al., 2006; Chan et al., 2010; Woo et al., 2012). Iron uptake in some prokaryotes is associated with *cirA2* (Uljanovas et al., 2023), which was highly upregulated in T1-21 but only slightly induced in the aerotolerant WP2202 strain. Since iron also functions in the formation of ROS, induction in the aerosensitive T1-21 strain render this strain more vulnerable to O_2_ exposure. Another gene involved in iron regulation is *dps,* which was mentioned earlier for its role in the oxidative stress response. Interestingly, *dps* differs from other iron-related genes in that it is upregulated at all timepoints in the aerotolerant WP2202 but is initially downregulated in the aerosensitive T1-21 strain. Since the Dps protein binds and sequesters iron, the aerotolerant strain may be recruiting this protein as a defense against iron-induced oxidative stress (Ishikawa et al., 2003).

A total of 17 ribosomal genes were downregulated in the T1-21 and WP2202 strains. Fifteen of these genes were more highly downregulated in the aerotolerant WP2202 strain than the aerosensitive T1-21. Ribosome biogenesis is quite costly to the bacterial cell (Zegarra et al., 2023), so expression of these genes may be downregulated when the bacterium is under stress as a means of conserving resources. Based on the differences in regulation of these genes in the two strains, the aerotolerant WP2202 is more effective at downregulating the expression of these genes, which could assist with its survival when it encounters O_2_.

Several genes were identified with contrasting regulation in the two *C. jejuni* strains. For example, *cheW*, *nrfA*, *tolQ2*, and *yknY* were upregulated in the aerosensitive T1-21 but downregulated in the aerotolerant WP2202 strain. Previous studies demonstrated that *cheW* mutants of *C. jejuni* more readily form biofilms as compared to wild-type strains (Tram et al., 2020). Furthermore, hyper-aerotolerant strains of *C. jejuni* were better at biofilm formation than other strains (Mouftah et al., 2021), suggesting that the aerotolerant WP2202 strain may downregulate *cheW* as a way of increasing its biofilm formation and enhancing tolerance to oxidative stress (Joshua et al., 2006; Asakura et al., 2007; Reuter et al., 2010; Turonova et al., 2015). It is unclear why the aerosensitive T1-21 strain induces *cheW* rather than downregulating it; however, *cheW* also has a role in chemotaxis (Reuter et al., 2020), and its upregulation may be part of an escape strategy for *C. jejuni*. The *nrfA* gene encodes a nitrite reductase that is involved in the nitrate respiration pathway, which allows nitrate to be used as an alternate electron acceptor when O_2_ is limiting (Pittman et al., 2007). The aerotolerant strain may downregulate this pathway under aerobic conditions when O_2_ is plentiful; however, it is unclear why *nrfA* was upregulated when O_2_ levels were high. Several genes in the aerotolerant strain (*tolQ2*, *yknY*) may be downregulated to more effectively manage resources under oxidative stress; however, it remains unclear why these genes were upregulated in the aerosensitive T1-21. In contrast to the previous genes discussed, *dcuA* was upregulated in the aerotolerant WP2202 strain but downregulated in the aerosensitive T1-21. In *E. coli*, *dcuA* encodes a membrane protein involved in transporting C4-dicarboxylates under anaerobic conditions (Six et al., 1994). Although *C. jejuni* does not grow well anaerobically, a prior study demonstrated that *dcuA* was upregulated in microaerobic conditions (Woodall et al., 2005). The aerosensitive T1-21 might downregulate *dcuA* in aerobic conditions during its transition to utilizing the aerobic C4-dicarboxylate transport system. It remains unclear why the aerotolerant WP2202 upregulated *dcuA* in aerobic conditions.

We hypothesized that the changes in gene expression associated with the oxidative stress response may occur rapidly after O_2_ exposure, and special note was taken of genes with large fold changes (magnitude > 10) at the 0.5 and 6 h timepoints. The genes with large spikes in gene expression at 0.5 or 6 h in the aerosensitive T1-21 included *btuB1*, *btuB2*, *cirA2*, *btuF*, *tpd*, *hmuU*, and *yknY*. The genes *cirA2*, *tpd*, and *hmuU* were discussed above for their roles in iron transport and metabolism, whereas *yknY* was mentioned due to its contrasting patterns of regulation in the two strains. The *btuB1*, *btuB2,* and *btuF* genes are involved in the acquisition of vitamin B12 in other bacterial species (Degnan et al., 2014; Poudel et al., 2023). Since B12 is critical for the functionality of many enzymes (Chimento et al., 2003), the high upregulation (fold changes ranging from 12 to 125) of these genes in the aerosensitive T1-21 strain may be a reaction to aerobic stress. Interestingly, WP2202 displayed only mild upregulation of *btuB1* and *btuB2* (fold changes < 10) and the data for *btuF* was not significant, indicating a less important role for these genes in the aerotolerant strain. Two unknown genes were also observed with high upregulation in the aerosensitive strain, including GenBank locus tags ASB61_RS03560 and a region overlapping locus tags ASB61_RS07715, ASB61_RS07720, and ASB61_RS07725. The locus ASB61_RS03560 was described in GenBank as “energy transducer TonB.” In *Campylobacter* spp., TonB proteins function in energy transduction from the inner to outer membrane, provide energy for the acquisition of ferric enterobactin (Zeng et al., 2013), and may also function in the oxidative stress response (Flint et al., 2014). It is tempting to speculate that locus ASB61_RS03560 encodes a gene that functions in iron acquisition in response to oxidative stress. The ASB61_RS07715, ASB61_RS07720, and ASB61_RS07725 loci are described in GenBank as a Fe-S protein, an ABC transporter permease, and a FtsX-like permease family protein. FtsX functions in the remodeling of bacterial cell walls during cell division (Mavrici et al., 2014), and it is possible that the aerosensitive T1-21 strain could upregulate this unknown gene in an attempt to reinforce the integrity of its cell wall during oxidative stress. Another unknown gene upregulated in T1-21 mapped to a region overlapping *chuA* and *chuB*, which are involved in heme uptake in *Campylobacter* (Ridley et al., 2006); this provides additional evidence that the aerosensitive strain upregulate iron acquisition genes during oxidative stress. Finally, an unknown gene on the pcjDM plasmid of the aerosensitive strain (locus tag ASB61_RS08465) was observed to be highly downregulated at 6 h. According to GenBank, this gene likely encodes a phage holin family protein, suggesting evidence of past phage infection. Since holin proteins are generally detrimental to bacteria (Young, 1992), it is reasonable to assume that this gene would be downregulated under most circumstances. The increase in downregulation of the gene under oxidative stress conditions as compared to microaerobic conditions might be evidence of the bacteria protecting itself from additional damage during stress.

Several genes that exhibited spikes in up- or down-regulation at the 0.5 or 6 h timepoints were present in the aerotolerant WP2202 strain as well, including *clpB*, *grpE*, *hrcA*, *katA*, and *kdgR*, and unknown genes with GenBank locus tags A0W69_06090, A0W69_06100, A0W69_08085, a region overlapping locus tags A0W69_08115 and A0W69_08120, A0W69_01600, and a region overlapping locus tags A0W69_08545 and A0W69_08550. Several of these genes were discussed above for their roles in heat shock (*clpB*, *hrcA*, *grpE*) or oxidative stress (*katA*). A spike in downregulation was observed at the 6 h timepoint for *kdgR* in the aerotolerant WP2202, but the data for *kdgR* expression in T1-21 was not significant. In other bacterial species, *kdgR* functions as a repressor in the metabolism of sugars (Pujic et al., 1998). Although *Campylobacter* was thought to lack carbohydrate metabolism, there is now evidence that it may have limited metabolism of carbohydrates (Garber et al., 2020); therefore, the downregulation of *kdgR* may be an attempt to use carbohydrates as an additional energy source during stress. The unknown gene with locus tag A0W69_01600 is highly downregulated at 6 h (fold change −141) and is described in GenBank as a “chemotaxis protein.” As discussed above, the chemotaxis gene *cheW* was somewhat downregulated in the aerotolerant WP2202. Since *cheW* mutants are better at biofilm formation (Tram et al., 2020), this gene may be downregulated for the purpose of biofilm formation and protection from oxidative stress. Two unknown genes, A0W69_06090 and A0W69_06100, have large amounts of downregulation (fold changes 11 and 12) at 0.5 h, however, both encode hypothetical proteins. An unknown gene (A0W69_08085) described as encoding a “fumarate reductase” was upregulated at 0.5 and 6 h. Oddly, fumarate reductases are thought to contribute to oxidative stress in bacteria via the formation of ROS (Messner and Imlay, 2002; Meehan and Malamy, 2012). Additionally, genes encoding fumarate reductases were upregulated when O_2_ was limiting in *Campylobacter* and are thought to function in anaerobic rather than aerobic respiration (Guccione et al., 2010). Further investigation is required to clarify the role of this enzyme during oxidative stress. Another unknown gene (overlapping locus tags A0W69_08115 and A0W69_08120) was described as a putative C4-dicarboxylate ABC transporter and was highly upregulated at 6 and 12 h. Similarly, the C4-dicarboxylate transporter gene *dcuA* was upregulated in the aerotolerant WP2202, suggesting that the aerotolerant strain may upregulate these transporters as a response to oxidative stress.

Several genes displayed spikes in up- or down-regulation at both the 12 and 24 h timepoints. In the aerosensitive strain, these included *acnB* and *ahpC* (possible oxidative stress genes), *clpB* and *hugZ* (iron acquisition or iron transport genes), and *tolQ2* and *nrfA*. Other genes with spikes in regulation at 12 or 24 h in the aerosensitive T1-21 strain include *flaC*, *mdtC*, *pfs*, *purL*, *secD*, *trpB, ttca,* and seven unknown genes on plasmid pcjDM (locus tags ASB61_RS08740, ASB61_RS08640, ASB61_RS08525, ASB61_RS08545, ASB61_RS08600, ASB61_RS08440, and ASB61_RS08430). In the aerotolerant strain, genes with spikes in regulation at 12 or 24 h included *atpC*, *dedA*, *ligA2*, *mmpA*, *modA*, *pth*, *purH*, *tal*, *tktA*, *torC*, and *torZ*. Many ribosomal genes were sharply downregulated at 12 or 24 h in one or both strains as well.

This study revealed several promising avenues for future research. Notably, the aerotolerant strain differentially regulated a greater number of genes at all timepoints than the aerosensitive strain, suggesting that it has a broader range of strategies for responding to oxidative stress. The spike in gene regulation commonly observed at 12 h in both strains is particularly intriguing and suggests that a potential shift in gene expression may occur at this time in response to prolonged oxidative stress. Further study of genes involved in iron acquisition that are upregulated in the aerosensitive T1-21 strain under aerobic conditions could reveal whether the phenomenon increases the incidence of iron-induced oxidative stress. Furthermore, the upregulation of heat shock genes in both strains was compelling, and further study of the connections between the oxidative and heat stress responses would be valuable. Additionally, the potential tendency of the aerotolerant strain to downregulate resource-intensive tasks warrants further study. One limitation of this study is the inclusion of only two strains of *C. jejuni*, which may not demonstrate the full range of genetic diversity in this species. Examining the gene expression of additional strains will be important to verify the patterns of expression observed. Moreover, transcriptomics can only tell us about changes in gene expression without fully illustrating the functional effects of each gene. Further evaluation in the form of knockout mutations or complementation studies will be required to verify the contribution of each gene to aerotolerance. This study builds on previous lines of research by providing additional data for the role of previously-characterized oxidative stress genes in the response of *C. jejuni* to aerobic conditions.

## Data Availability

The datasets presented in this study can be found in online repositories. The names of the repository/repositories and accession number(s) can be found in the article/Supplementary material.

## Footnotes

^1^ http://eggnog-mapper.embl.de/
