# Supplementary material for: Transcriptome analysis of aerotolerant and aerosensitive Campylobacter jejuni strains in aerobic conditions
Source: Front Microbiol. 2025 Jul 30;16:1621314. doi: 10.3389/fmicb.2025.1621314 (PMC12343642; doi:10.3389/fmicb.2025.1621314)
Supplement: Supplementary file 2 [file Image_1.pdf]

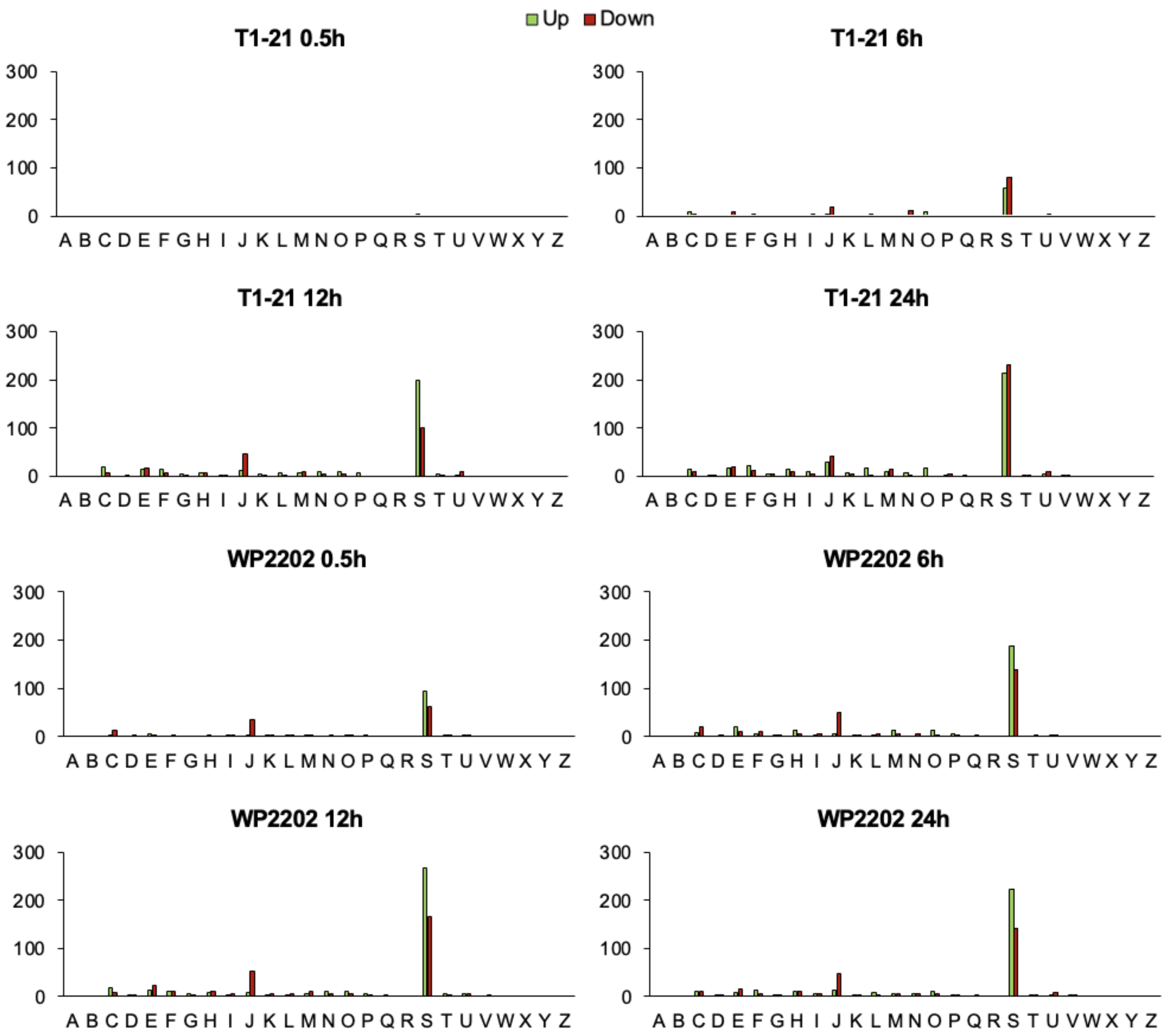

FIGURE S1: Number of genes significantly up or down-regulated in different COG categories. Plasmid genes are excluded. Data represent gene regulation in the aerosensitive *C. jejuni* strain T1-21 and the aerotolerant strain WP2202 as a function of time. Significance was defined as FDR *P*-values less than 0.05 and fold-change magnitudes of 1.5 or greater.
